# Breaking the malaria barrier: the WHO-approved R21/Matrix-M vaccine and its global impact – an editorial

**DOI:** 10.1097/MS9.0000000000001648

**Published:** 2024-03-04

**Authors:** Amogh Verma, Ayush Anand, Vaishnavi A. Patel, Muhammad W. Nazar, Ankini Mukherjee, Karim A. Karim, Malik O. Oduoye, Prakasini Satapathy, Sarvesh Rustagi

**Affiliations:** a Rama Medical College Hospital and Research Centre, Hapur; b Center for Global Health Research, Saveetha Medical College and Hospital, Saveetha Institute of Medical and Technical Sciences, Saveetha University, Chennai; c GMERS Medical College and Hospital, Gotri; d School of Applied and Life Sciences, Uttaranchal University, Uttarakhand, India; e Medical Laboratories Techniques Department, AL-Mustaqbal University, Hillah, Babil, Iraq; f Rampurhat Government Medical College and Hospital, Rampurhat, India; g Ahmadu Bello University, Zaria, Nigeria; h Kamuzu University of Health Sciences, Blantyre, Malawi; i CMH Multan Institute of Medical Sciences, Multan, Pakistan; j BP Koirala Institute of Health Sciences, Dharan, Nepal

*Dear Editor*,

According to a recent study by the WHO in late 2021, malaria claimed ~627 000 lives in 2020, with a child mortality rate rising from 4.8 to 7.8%^1^. The burden of malaria reached 1.7 billion total cases and 10.6 million deaths in that year alone^1^. The disease’s impact varies from one country to another, with 11 countries carrying over 70% of the global malaria burden^1^. Malaria remains a significant public health concern in India and globally^1^. Although WHO reports a decrease in the number of cases in India, from 20 million in 2000 to ~5.6 million in 2019, the disease continues to affect pregnant mothers and neonates, leading to mild to severe anemia in mothers and significant impacts on neonatal health, such as stunted growth and low birth weight^2^.

## Need for newer vaccines for malaria control

Despite its global impact, the *Plasmodium vivax* malaria parasite has long been neglected in vaccine development^3^. The first developed vaccine, RTS,S, proved ineffective against the *P. vivax* species and faced resistance to antimalarial drugs, such as chloroquine and primaquine. Additionally, the parasite has the unique ability to persist in the liver, causing asymptomatic malaria^3^. To mitigate the burden of malaria, the first malaria vaccine, RTS,S, was developed after extensive trials and proved effective primarily for small children in highly endemic areas^4^. However, it had several limitations due to a poor understanding of the immunogenic mechanisms against malaria and the phenomenon of rebound malaria^4^. The vaccine only targeted sporozoites, delaying the acquisition of natural immunity, which led to malaria symptoms returning after its effect wore off^5^. The R21/Matrix-M vaccine is the second WHO-recommended vaccine, following the RTS,S vaccine, which had undergone several phases of clinical trials^5^. WHO has noted the increased demand for the RTS,S malaria vaccine and the need for a second one to provide faster protection for children, thereby advancing the dream of a malaria-free community^5^.

## Safety and efficacy profile of R21/Matrix-M malaria vaccine

**Figure 1:**
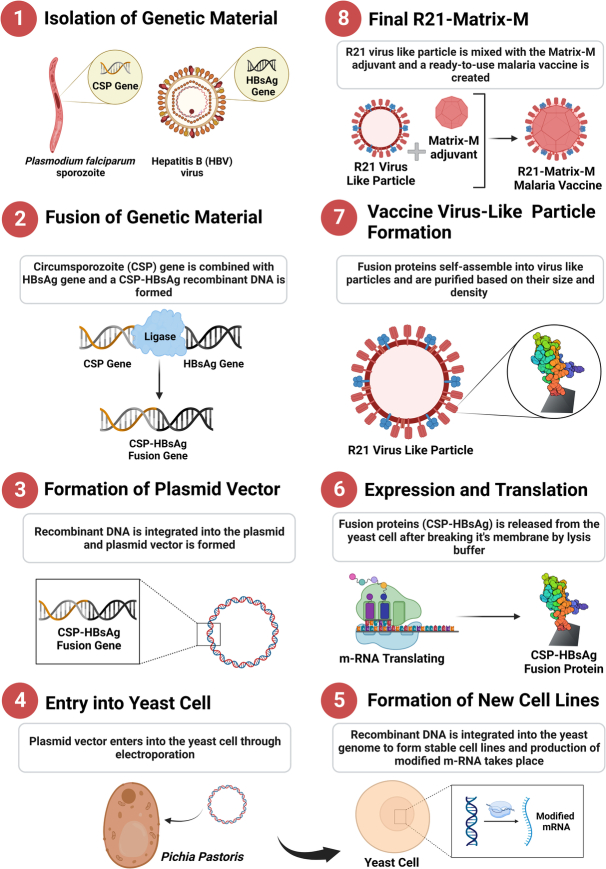
Malaria (R21) investigational vaccine production process [Created with Biorender.com].

The newly developed R21/Matrix-M malaria vaccine represents a significant milestone in the fight against malaria. Developed as the successor to the RTS,S/AS01 (RTS,S) vaccine by the University of Oxford and the Serum Institute of India (SII), the R21/Matrix-M leverages Novavax’s Matrix-M adjuvant technology (Fig. 1) demonstrates high efficacy with a reassuring safety profile^6^. In a pivotal large-scale Phase III clinical trial, the vaccine has been administered to 4800 children across four African countries, including Burkina Faso, Kenya, Mali, and Tanzania^6^. However, the Phase III trial results are under clinical observation before publication^6^.

The R21/Matrix-M malaria vaccine has shown safety and high effectiveness across multiple clinical studies^5^. It is easily transferable, cost-effective, and readily available for distribution, particularly in African countries where malaria poses a significant threat to public health^5^. The groundbreaking R21/Matrix-M malaria vaccine results from innovative research at the Jenner Institute, Oxford University^6,7^. Developed in 2012 as an improved version of RTS,S, the R21 vaccine features a redesigned Hepatitis B surface antigen fusion, enhancing particle formation by eliminating excess unfused HBsAg^7^. This modification increases the density of Plasmodium falciparum circumsporozoite protein (CSP) antigen on the virus-like particle^7^. The vaccine, produced using the yeast expression system Pichia pastoris, contains ~25 μg of CSP in a 50 μg R21 dose, compared to 10 μg in RTS,S^7^. The Oxford researchers played a pivotal role in designing the vaccine, while the introduction of Novavax’s Matrix-M as an adjuvant significantly boosted the vaccine’s immune response and efficacy^8^. With the SII’s commitment to mass production, this collaborative effort is poised to address the severe malaria burden, particularly in Africa^9^.

In a pivotal Phase III trial led by the SII, the WHO-endorsed R21/Matrix-M vaccine demonstrated promising results^10^. It achieved a 12-month efficacy of 75% in seasonal areas and 68% in perennial regions, with a booster dose restoring efficacy to 74% over 18 months, particularly in seasonal sites^10^. The 5–17-month age group exhibited higher effectiveness, with 79% efficacy in seasonal and 75% in standard settings^10^. Furthermore, the vaccine’s safety profile was favorable, with mild adverse events noted^10^. However, it is important also to consider the limitations of this innovative vaccine, which are summarized in Table 1 ^9^. The vaccine’s primary efficacy analysis at 6 months revealed 74% efficacy in group 1 and 77% in group 2^10^. At 1 year, efficacy remained high at 77% in group 1, and post-third vaccination, participants displayed elevated anti-NANP antibody titers, which nearly doubled with a higher adjuvant dose^10^. Despite waning, titers were boosted to peak levels after a fourth dose 1 year later^10^.

**Table 1:** Limitations of R21/Matrix-M malaria vaccine

Limitation	Description
Limited generalizability	The study’s findings may not extend to populations beyond the African nations where the vaccine was exclusively tested, as the R21/Matrix-M vaccine was evaluated solely in specific African regions and may not represent a global population
Efficacy on *P. falciparum* only	This vaccine’s effectiveness is confined to Plasmodium falciparum, one of the malaria parasites, providing no protection against other malaria parasites like Plasmodium vivax, prevalent in different parts of the world
Safety concerns of RTS,S/AS01	Previous concerns with the RTS,S/AS01 malaria vaccine, including an increased incidence of meningitis and cerebral malaria cases, raise questions about the safety profile of the new R21/Matrix-M vaccine
Limited follow-up	Assessment of the vaccine’s efficacy over a relatively short follow-up period may potentially fail to capture its long-term effectiveness or the potential waning of protection over time
Possible bias	Potential biases, such as the selection of the study population or the timing of vaccine administration in relation to the malaria season, could introduce variability in the outcomes
Incomplete immune response understanding	While the vaccine induced robust antibody responses to specific antigens, it remains unclear whether these responses correlate with complete protection against malaria or if other immune factors play a role
Uncertainty about long-term protection	Lack of information on the vaccine’s efficacy beyond the 12-month mark is crucial for understanding its long-term protective effects
Limited discussion on feasibility	Mention of concerns regarding the feasibility of a four-dose schedule without thorough exploration of this aspect may potentially impact the practicality of vaccine deployment
Exclusion of specific age groups	The primary focus on children aged 5–17 months may potentially exclude other age groups, limiting the broader applicability of the vaccine
Lack of comparative data	Absence of a direct comparison of the R21/Matrix-M vaccine’s efficacy with the previous RTS,S/AS01 vaccine makes it challenging to assess whether it offers a substantial improvement
Lack of real-world data	While the provision of data from controlled human malaria infection trials is valuable, the real-world efficacy and impact of the vaccine in a natural setting remain uncertain

The R21/Matrix-M vaccine, with the Matrix-M adjuvant, has exhibited robust antibody responses and promising sterile efficacy rates of 63–78% in controlled human malaria infection trials^11^. The WHO reports that the R21 vaccine reduced symptomatic malaria cases by 75% in the 12 months following a 3-dose series, with efficacy sustained by a fourth dose a year later^11^.

SII plays a crucial role in amplifying the global impact of the R21/Matrix-M malaria vaccine^9^. SII has established a production capacity of 100 million doses annually, set to double in the next 2 years, making this vaccine an easily deployable solution^9^. With a price range of $2 to $4 per dose and a four-dose requirement per person, the R21/Matrix-M vaccine offers a cost-effective advantage, roughly half the cost of the RTS,S vaccine. This significant-scale production ensures widespread availability and lower per-unit costs, enhancing affordability^5^. Despite the low cost and efficacy, high temperature sensitivity and photosensitivity of R21/Matrix-M vaccine mandates for improved cold chain facilities and is a major limitation for distribution of vaccine in remote areas^9^.

Through mathematical modeling, Nora *et al*.^11^ assessed the potential impact and cost-effectiveness of implementing the R21/Matrix-M vaccine. This study considered various malaria transmission settings, particularly in sub-Saharan Africa, and assessed the effects of vaccine deployment^11^. The results indicated that incorporating the vaccine into routine childhood immunization programs could significantly reduce malaria cases and deaths among children in malaria-prone areas across sub-Saharan Africa^11^.

## Recommendations to mitigate malaria

Several essential steps are required to achieve success in the battle against malaria. Firstly, increasing the efficiency of light microscopes is imperative, as a significant portion of malarial parasites exhibits sub-microscopic characteristics (3.6% in India), rendering them undetectable through conventional methods^3^. Thus, the development and implementation of enhanced detection techniques are vital^3^. Secondly, the presence of experienced laboratory workers is crucial, particularly in regions with a high prevalence of G6PD deficiency^12^. Skilled technicians play a pivotal role in the early detection of enzyme deficiencies, ensuring the safe initiation of antimalarial therapy^3^. Furthermore, continued research is necessary to understand the impact of COVID-19 on the malaria burden and identify the factors contributing to the substantial reduction in malaria cases^3^. This ongoing research is fundamental to achieving the ambitious goal of malaria eradication by 2030^3^. Lastly, establishing robust surveillance systems is essential in high-endemic areas like the Himalayan regions in Uttarakhand, India^3^. Such systems aid in effectively combating seasonal malaria and educating communities about the disease and its prevention^3^. Another critical aspect of successful vaccination program is community acceptance and logistic of adequate delivery^13,14^. It is necessary to ensure proper education regarding vaccine and its side effects, involvement of community in vaccination, and adequate healthcare services to manage the adverse effects^12–15^. In addition, further research is mandated to develop vaccines for malaria. As per WHO, 89 malaria vaccines and 153 clinical trials have been completed or are active^16^. Of this 29 vaccines are in active stage and 25 clinical trials are ongoing^16^.

## Conclusion

R21/Matrix-M malaria vaccine represents a significant leap forward in the global fight against malaria. With its high efficacy, affordability, and potential to save countless lives, it offers a beacon of hope in reducing the burden of this devastating disease and moving us closer to a malaria-free world. However, it is vital to remember that the fight against malaria is multifaceted, and ongoing research and efforts beyond the vaccine are crucial to achieving the ambitious goal of malaria eradication. In the face of this complex challenge, the R21/Matrix-M vaccine is a powerful tool in our arsenal, illuminating a path towards a malaria-free future.

## Ethical approval

Ethics approval was not required for this editorial article.

## Consent

Informed consent was not required for this editorial article.

## Sources of funding

The authors did not receive any funding for this work.

## Author contribution

A.V.: conceptualization, supervision, writing – original draft, and writing – review and editing; A.A.: supervision, writing – original draft, and writing – review and editing; V.A.P., M.W.N., A.M., K.A.K., M.O.O., P.S., and S.R.: writing –original draft, writing – review and editing. All authors approve the final version of the manuscript.

## Conflicts of interest disclosures

The authors declare no conflicts of interest to declare.

## Research registration unique identifying number (UIN)

Not applicable.

## Guarantor

Amogh Verma.

## Data availability statement

Data sharing is not applicable to this article.

## Assistance with the study

Not applicable.
